# Facilitation of Insulin Effects by Ranolazine in Astrocytes in Primary Culture

**DOI:** 10.3390/ijms231911969

**Published:** 2022-10-09

**Authors:** Adrián Jordá, Martin Aldasoro, Ignacio Campo-Palacio, Jose M. Vila, Constanza Aldasoro, Juan Campos-Campos, Carlos Colmena, Sandeep Kumar Singh, Elena Obrador, Soraya L. Valles

**Affiliations:** 1 Department of Physiology, University of Valencia, 46010 Valencia, Spain; adrian.jorda@uv.es (A.J.); martin.aldasoro@uv.es (M.A.); igcampa@alumni.uv.es (I.C.-P.); vila@uv.es (J.M.V.); constanza.aldasoro@gmail.com (C.A.); juan.campos-campos@uv.es (J.C.-C.); carcarmafisio@gmail.com (C.C.); elena.obrador@uv.es (E.O.); 2 Department of Nursing and Podiatry, University of Valencia, 46010 Valencia, Spain; 3 Indian Scientific Education and Technology Foundation, Lucknow 226002, India

**Keywords:** ranolazine, insulin, astrocytes, inflammation, antioxidants

## Abstract

Ranolazine (Rn) is a drug used to treat persistent chronic coronary ischemia. It has also been shown to have therapeutic benefits on the central nervous system and an anti-diabetic effect by lowering blood glucose levels; however, no effects of Rn on cellular sensitivity to insulin (Ins) have been demonstrated yet. The present study aimed to investigate the permissive effects of Rn on the actions of Ins in astrocytes in primary culture. Ins (10^−8^ M), Rn (10^−6^ M), and Ins + Rn (10^−8^ M and 10^−6^ M, respectively) were added to astrocytes for 24 h. In comparison to control cells, Rn and/or Ins caused modifications in cell viability and proliferation. Rn increased protein expression of Cu/Zn-SOD and the pro-inflammatory protein COX-2 was upregulated by Ins. On the contrary, no significant changes were found in the protein expression of NF-κB and IκB. The presence of Rn produced an increase in p-ERK protein and a significant decrease in COX-2 protein expression. Furthermore, Rn significantly increased the effects of Ins on the expression of p-AKT, p-eNOS, p-ERK, Mn-SOD, and PPAR-γ. In addition, Rn + Ins produced a significant decrease in COX-2 expression. In conclusion, Rn facilitated the effects of insulin on the p-AKT, p-eNOS, p-ERK, Mn-SOD, and PPAR-γ signaling pathways, as well as on the anti-inflammatory and antioxidant effects of the hormone.

## 1. Introduction

Astrocytes are the most abundant cells in the central nervous system (CNS) and perform a variety of functions, including structural support, blood–brain barrier integrity, and the development of important protective roles [1]. They also take part in immunological responses and in the reparative processes that occur at different stages of neuroinflammation [2].

Astrocytes secrete both neurotrophic and inflammatory cytokines, and express receptors for mediators such as Interleukin-1β (IL-1β) and Tumor Necrosis Factor-α (TNF-α), among others [3,4]. Glucose absorption and storage are two of insulin’s most essential effects [5]. Insulin crosses the blood–brain barrier, acting on astrocytes and, indirectly, on neurons [6]. The brain expresses insulin receptors (IRs) on neurons, microglia, and astrocytes. Its effects include metabolic functions and neuronal survival after trauma or during neurodegeneration [7]. In fact, these effects are due to anti-inflammatory insulin action. At 10^−8^ M, insulin inhibits inducible nitric oxide synthase (iNOS) expression and NF-κB (Nuclear Factor-κB) level increase in astrocytes induced by Bacterial Lipopolysaccharide (LPS) [8]. Furthermore, insulin increased the vitality of rat and human astrocytes [9,10]. Insulin is generally degraded in lysosomes within cells [6], although there is evidence of the presence of the insulin-degrading enzyme (IDE) in different types of cells, including astrocytes [11]. In addition, IDE degrades other peptides such as a beta-amyloid peptide, which is involved in the pathogenesis of Alzheimer’s disease (AD) [12]. Astrocytes take up glutamate at the synapse, triggering aerobic glycolysis and a secretion of lactate, which can be used by neurons as a source of energy after activity [13,14]. In astrocytes, insulin increases glycogen storage, stimulates glucose uptake through GLUT4, and modulates the inflammatory response [15,16]. Interestingly, activation of insulin-mediated pathways was downregulated in astrocytes in response to chronically elevated insulin levels, but not in neurons [17]. These cellular differences could have implications for the effects of T2D (type 2 diabetes) and insulin resistance on the function of different types of brain cells.

In clinical practice, ranolazine (Rn) is used to treat refractory chronic stable angina [18,19]. Data from patients indicate that ranolazine preserves myocardial blood flow during ischemic insults [20]. Human studies back up the idea that ranolazine can help improve coronary blood flow by lowering the mechanical consequences of ischemia contracture, enhancing endothelial function, or both [21,22]. At therapeutic concentrations, Rn inhibits the late inward sodium current (I(NaL)) [23], reducing tissue damage caused by intracellular sodium and calcium overload, which is associated with myocardial ischemia [24,25,26]. I(NaL) amplitude is increased in many pathological situations, such as myocardial ischemia and oxidative stress [27,28,29]. In addition to its antianginal effects, Rn acts as an anti-inflammatory agent, reducing asymmetric dimethylarginine and C-reactive protein plasma levels and promoting the endothelial release of vasodilator mediators in patients with ischemic coronary disease [30]. Furthermore, metabolic effects, such as the lowering of hemoglobin A1c (HbA1c) in patients with ischemic heart disease and diabetes [31,32,33], or the improvement of insulin secretion and β-cell survival in diabetic mice [34], have already been described. Moreover, several studies evaluated the effects of Rn on the cardiovascular [35,36,37] and nervous systems [4,38,39]. They suggested that these effects would also be mediated by late INa or inwardly rectifying K^+^ current [40]. 

Therefore, the objective of this study is to evaluate the effects of insulin on astrocytes in primary culture and the facilitating actions of ranolazine on the sensitivity of astrocytes to insulin (Ins). It is intended to evaluate the effects of insulin and ranolazine (Rn) on cell viability, as well as on anti-inflammatory and antioxidant mechanisms and processes.

## 2. Results

### 2.1. Cell Viability

**Figure 1:**
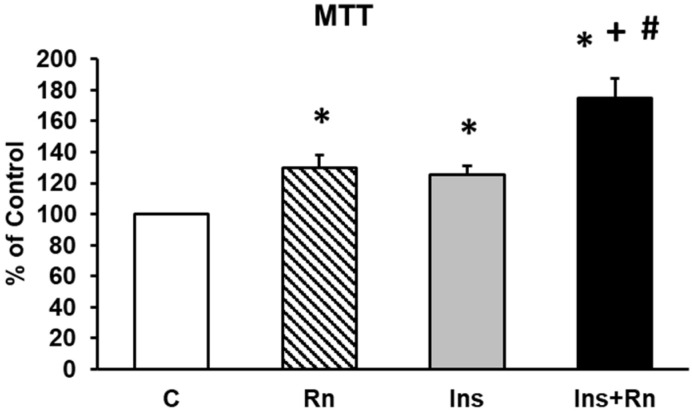
Effect of Ins and Rn on astrocytes in primary culture. Cell viability was determined by MTT assay in cells treated for 24 h. Astrocytes were incubated without Ins or Rn (control, C), with Rn (10^−6^ M), with Ins (10^−8^ M), or with Ins + Rn (10^−6^ M + 10^−8^ M). Data are mean ± SD of four independent experiments (four different rats). * *p* < 0.05 vs. control. + *p* < 0.05 vs. Rn. # *p* < 0.05 vs. Ins.

The roles of Rn, Ins, and Ins + Rn on astrocytes’ viability were studied using an MTT conversion assay. Figure 1 shows the viability of astrocytes in primary culture. The incubation with Rn, Ins, and Ins + Rn, produced significant increases compared with control astrocytes (Figure 1) (Rn 28%, Ins 27%, and Ins + Rn 72%). Furthermore, Ins + Rn produced an increase in viability compared to Ins of about 25%.

**Figure 2:**
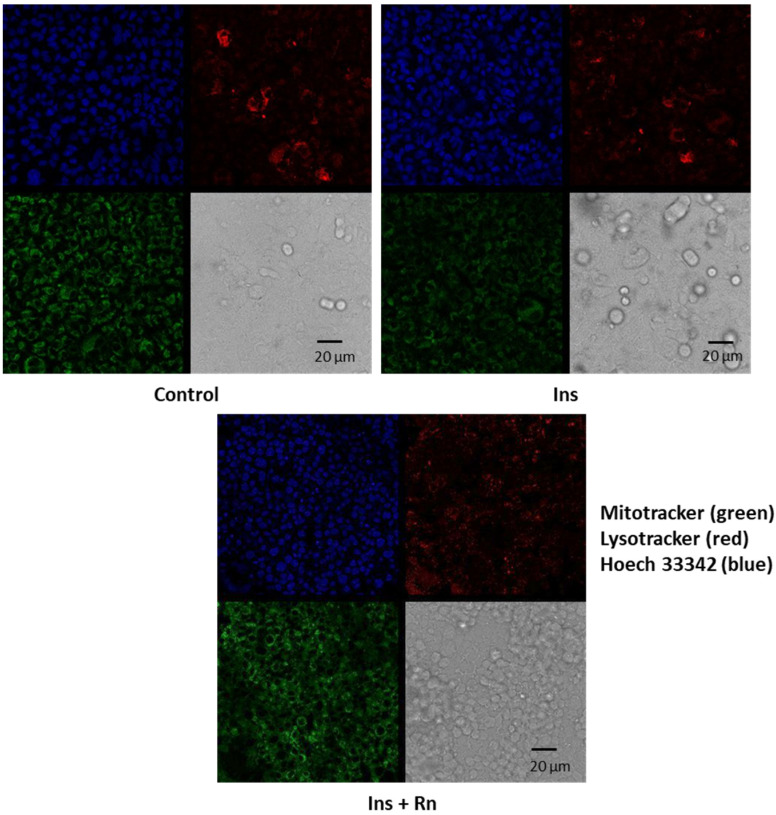
Effect of Ins and Ins + Rn on astrocytes in primary culture. Cells were isolated and seeded at 7 × 10^4^ cells/35 mm dish for 5 days. Cells were incubated without Ins or Rn (control, C), with Ins (10^−8^ M), or with Ins + Rn (10^−8^ + 10^−6^ M) for 24 h. Fluorescence products used were: Mitotracker (250 nM) (in green) to stain mitochondria, Lysotracker (250 nM) (in red) to stain lysosomes, and Hoechst 33342 (2 μg mL^−1^) (in blue) to stain nuclei. Contrast images are added. Bar represents 20 μm.

Figure 2 shows that Ins and Ins + Rn increased astrocytes numbers more compared to control cells. Cells were isolated and seeded at 7 × 10^4^ cells/35 mm dish for 5 days, and afterwards we added Ins or Ins + Rn for 24 h. Fluorescence with Mitotracker to stain mitochondria, lysotracker to stain lysosomes, and Hoechst 33342 to stain nuclei were used to show changes in the numbers of mitochondria, lysosomes, and nuclei. 

### 2.2. Cell Proliferation

A trypan blue exclusion assay was used to count the living cells and monitor cell proliferation. Astrocytes were isolated and seeded at 7 × 10^4^ cells/35 mm dish. After 5 days of culture, cells were incubated without Ins or Rn (control, C) or with Rn (10^−6^ M), Ins (10^−8^ M), or Ins + Rn (10^−8^ and 10^−6^ M) for 24 h. In control conditions, proliferation was 0.85%; with Rn, 30.31%; with Ins, 29.18% and with Ins + Rn, 33.91%, demonstrating significant differences (Table 1). 

### 2.3. Protein Expression of p-AKT 

Protein kinase B (PKB) is a family of proteins encoded in humans by three genes: AKT1, AKT2, and AKT3. AKT2 is an important signaling molecule in the insulin signaling pathway (induces glucose transport). When the insulin receptor (IR) is activated, the PI3K (phosphatidylinositol 3-kinase) is activated, which will phosphorylate PIP2 (phosphatidylinositol 3,4-bisphophate) to form PIP3 (phosphatidylinositol 3,4,5-triphosphate) [41]. In patients with type 2 diabetes, there is interference in the intracellular signaling pathway of PI3K/PKB produced by increases in cAMP [42]. Thus, we will determine the changes in protein expression after Ins addition to astrocytes in primary culture.

**Figure 3:**
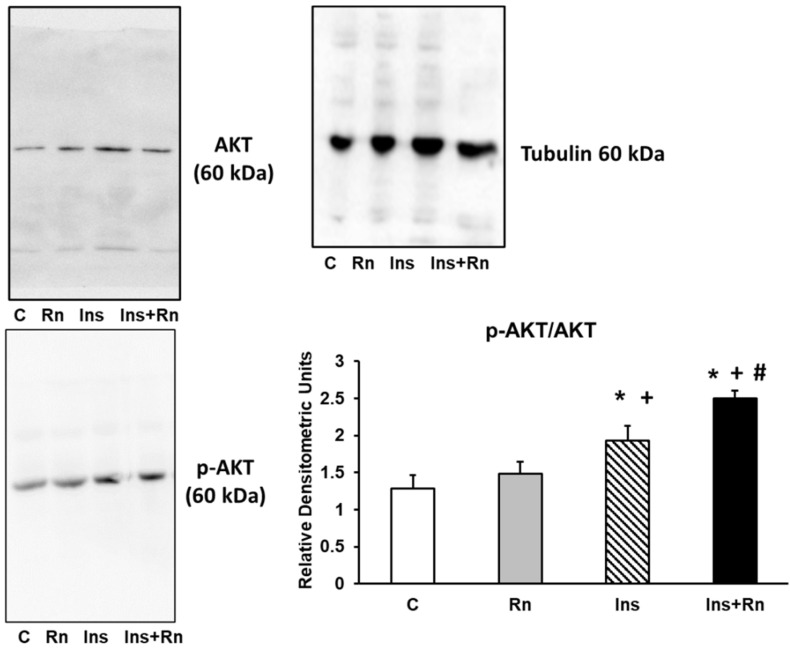
Effect of Ins and Rn on p-AKT and AKT protein expression. Astrocytes were incubated without Ins or Rn (control, C), with Rn (10^−6^ M), with Ins (10^−8^ M), or with Ins + Rn (10^−8^ M + 10^−6^ M) for 24 h and collected to determine p-AKT (1:500), AKT (1:500), and Tubulin (1:3000) protein expressions by Western blot. A representative immunoblot is shown in the panel. Data are mean ± SD of four independent experiments (four different rats). * *p* < 0.05 vs. control. + *p* < 0.05 vs. Rn. # *p* < 0.05 vs. Ins.

Figure 3 shows that Rn (10^−6^ M), Ins (10^−8^ M), and Ins + Rn (10^−8^ M and 10^−6^ M) produced significant differences in p-AKT compared to control cells. In fact, Ins increased the expression of p-AKT by 43.3% compared to the control and Ins + Rn increased by 87.2% compared to the control. Furthermore, Rn did not produce significant changes compared to control cells. In addition, the joint effect of Ins + Rn increased the expression of p-AKT with respect to Ins by 31.6% (Figure 3). 

### 2.4. Expression of p-eNOS Protein

Endothelial NOS (eNOS) synthesize nitric oxide (NO). Impaired NO production is involved in the pathogenesis of several diseases such as hypertension [43], diabetes mellitus [44], and migraine [45].

**Figure 4:**
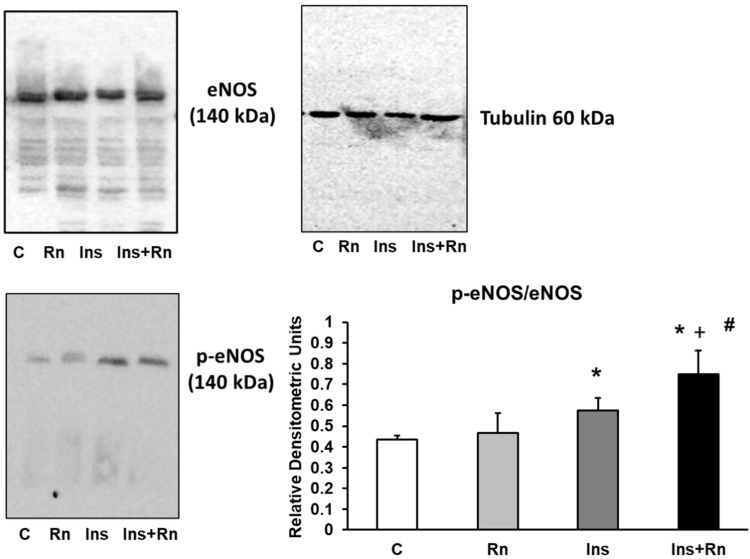
Effect of Ins and Rn on p-eNOS and eNos protein expression. Astrocytes were incubated without Ins or Rn (control, C), with Rn (10^−6^ M), with Ins (10^−8^ M), or with Ins + Rn (10^−8^ M + 10^−6^ M) for 24 h and collected to determine p-eNOS (1:250), eNOS (1:250), and Tubulin (1:3000) protein expressions by Western blot. A representative immunoblot is shown in the panel. Data are mean ± SD of four independent experiments (four different rats). * *p* < 0.05 vs. control. + *p* < 0.05 vs. Rn. # *p* < 0.05 vs. Ins.

Here, we determined the expression of p-eNOS in astrocytes in primary culture. The presence of Rn did not produce any significant differences in respect to control cells. Ins increased the expression of p-eNOS protein compared to control cells (32.25%). Ins + Rn significantly increased the expression of p-eNOS compared to the control (74.1%). Furthermore, the joint effect of Ins + Rn significantly increased (30.6%) the expression of p-eNOS with respect to the Ins group (Figure 4).

### 2.5. p-ERK Protein Expression

Extracellular signal-regulated kinase 1/2 (ERK) belongs to the mitogen-activated protein kinase (MAPK) family, which plays a role in signaling cascades and transmits extracellular signals to intracellular targets. Moreover, ERK exists as isozymes including ERK I (44 kDa) and ERK II (42 kDa). Insulin induces a rapid translocation of the MEK from the cytoplasm to the nucleus and activates resident nuclear ERK I/II in cells [46].

**Figure 5:**
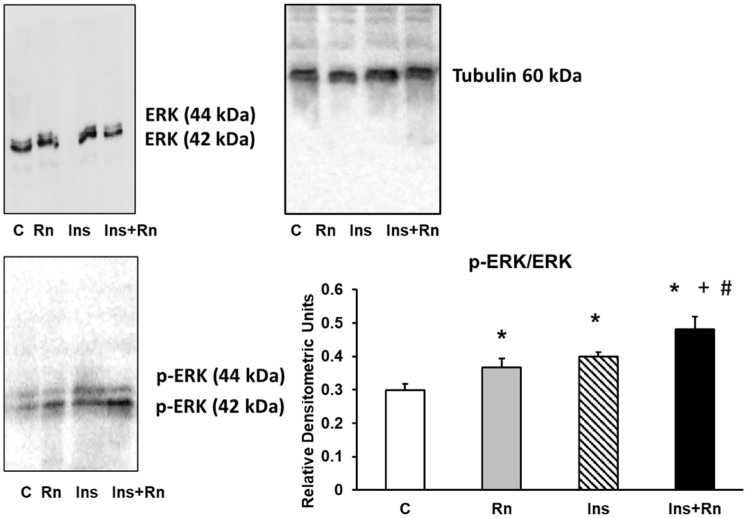
Effect of Ins and Rn on p-ERK and ERK protein expression. Astrocytes were incubated without Ins or Rn (control, C), with Rn (10^−6^ M), with Ins (10^−8^ M), or with Ins + Rn (10^−8^ M + 10^−6^ M) for 24 h and collected to determine p-ERK (1:500), ERK (1:500), and Tubulin (1:3000) protein expressions by Western blot. A representative immunoblot is shown in the panel. Data are mean ± SD of four independent experiments (four different rats). * *p* < 0.05 vs. control. + *p* < 0.05 vs. Rn. # *p* < 0.05 vs. Ins.

We determined p-ERK protein expression in astrocytes in primary culture. After addition of Rn or Ins, a significant increase in p-ERK protein expression was detected compared to control astrocytes (22.8% and 33.2%). The incubation with Ins + Rn significantly increased p-ERK expression compared to control cells (60.1%) and in respect to Rn- or Ins-treated cells (29.7 and 21.4%, respectively) (Figure 5). 

### 2.6. COX-2 Protein Expression

Inducible cyclooxygenase (COX-2) is expressed under inflammatory conditions and COX-2 inhibition can potentially develop into a preventive therapy against diabetes mellitus [47].

**Figure 6:**
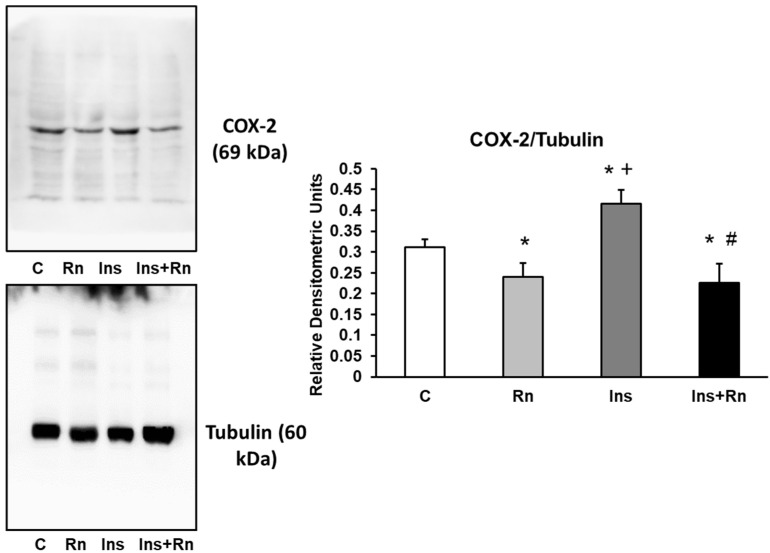
Effect of Ins and Rn on COX-2 protein expression. Astrocytes were incubated without Ins or Rn (control, C), with Rn (10^−6^ M), with Ins (10^−8^ M), or with Ins + Rn (10^−8^ M + 10^−6^ M) for 24 h and collected to determine COX-2 (1:500) and Tubulin (1:3000) protein expression by Western blot. A representative immunoblot is shown in the panel. Data are mean ± SD of four independent experiments (four different rats). * *p* < 0.05 vs. control. + *p* < 0.05 vs. Rn. # *p* < 0.05 vs. Ins.

We detected a significant decrease after addition of Rn (10^−6^ M) and an increase in COX-2 protein expression after addition of Ins (10^−8^ M) compared with control values (15.2% and 20.1%, respectively). Furthermore, the presence of Ins + Rn decreased COX-2 expression (18.1%) in respect to control astrocytes and 48.8% in respect to astrocytes treated with Ins, showing no differences in respect to Rn addition (Figure 6).

### 2.7. Expression of Cu/Zn-SOD and Mn-SOD Proteins

The enzyme superoxide dismutase (SOD) catalyzes the dismutation of superoxide into oxygen and hydrogen peroxide and is an important antioxidant defense in most cells exposed to oxygen [48]. Genetic disruption of the SOD1 gene causes glucose intolerance and impairs β-cell function [49].

**Figure 7:**
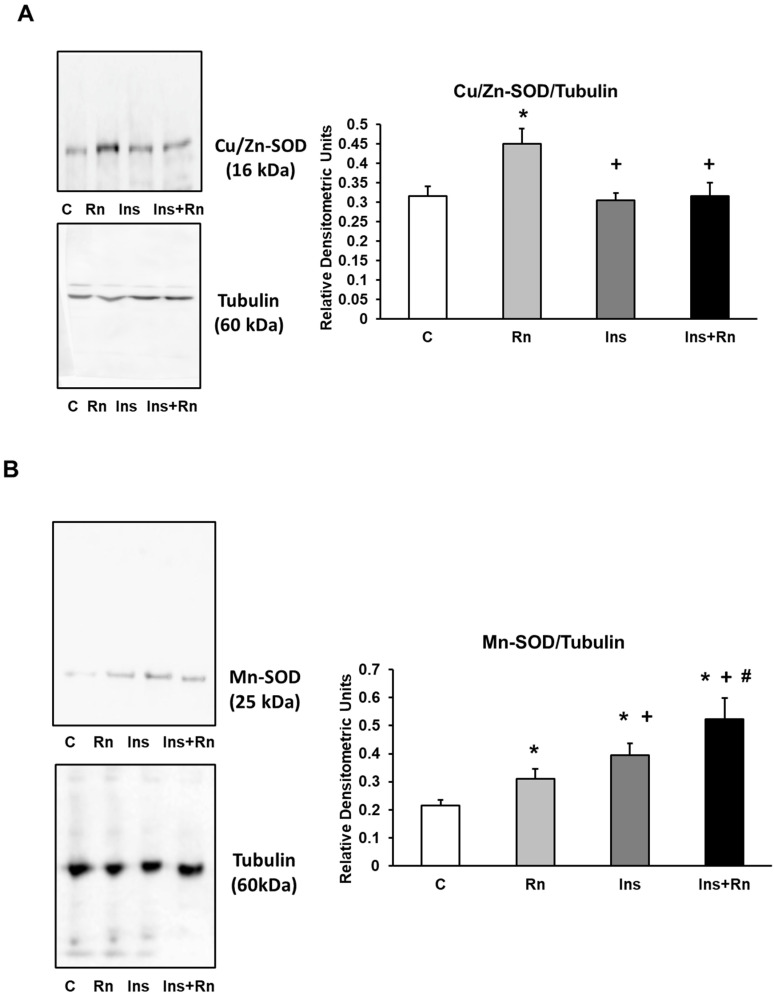
Effect of Ins and Rn on Cu/Zn-SOD (**A**) and Mn-SOD (**B**) protein expression. Astrocytes were incubated without Ins or Rn (control, C), with Rn (10^−6^ M), with Ins (10^−8^ M), or with Ins + Rn (10^−8^ M + 10^−6^ M) for 24 h and collected to determine Cu/Zn-SOD (1:500) (**A**) and Mn-SOD (1:250) (**B**), protein expression by Western blot. Tubulin (1:3000) was added in both figures as loading control. Representative immunoblots are shown in the panels. Data are mean ± SD of six independent experiments (six different rats). * *p* < 0.05 vs. control. + *p* < 0.05 vs. Rn. # *p* < 0.05 vs. Ins.

In astrocytes, Rn produced changes in Cu/Zn-SOD, but Ins and Ins + Rn did not produce changes in comparison to control cells (Figure 7A). Expression of Mn-SOD was determined and is shown in Figure 7B. The addition of Rn and Ins significantly increased protein expression compared to control astrocytes (30.1% and 51.2%, respectively). Incubation with Ins + Rn also significantly increased Mn-SOD protein expression compared to the control (59.1%), Rn (58.9%), and Ins (16.4%) (Figure 7B).

### 2.8. NF-κB and IκB Expression

**Figure 8:**
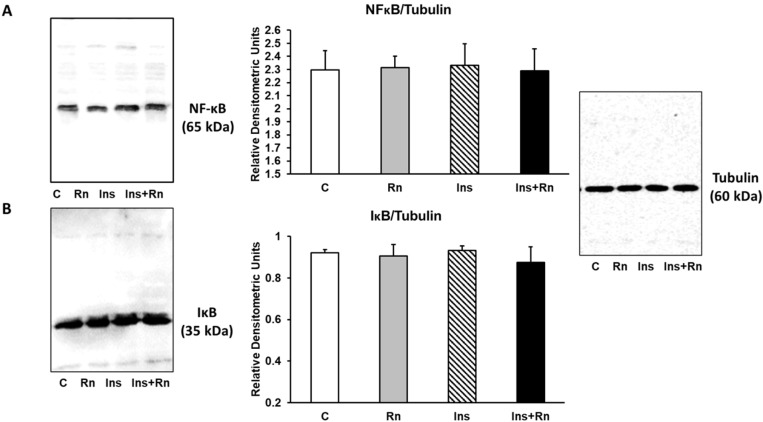
Effect of Ins and Rn on NFκB (**A**) and IκB (**B**) protein expression. Astrocytes were incubated without Ins or Rn (control, C), with Rn (10^−6^ M), with Ins (10^−8^ M), or with Ins + Rn (10^−8^ M + 10^−6^ M) for 24 h and collected to determine NFκB (1:250) (**A**) and IκB (1:250) (**B**) protein expression by Western-blot. Tubulin (1:3000) was added in both figures as loading control). Representative immunoblots are shown in the panels. Data are mean ± SD of four independent experiments (four different rats).

NF-κB is a transcription factor that regulates the positive gene expression of pro-inflammatory proteins. Figure 8A shows that Rn (10^−6^ M), Ins (10^−8^ M), and Ins + Rn (10^−8^ M and 10^−6^ M) did not produce significant differences compared to control cells. Moreover, IκB is one member of a family of cellular proteins that inhibit the NF-κB transcription factor. When the separation of IκB and NF-κB occurs, IκB is destroyed and NF-κB enters the nucleus and binds to DNA.

Figure 8B shows that Rn, Ins, and Ins + Rn did not induce significant differences in IκB protein expression compared to control cells. No change in expression of either protein was detected indicating that NF-κB activation did not occur.

### 2.9. PPAR-γ Expression

**Figure 9:**
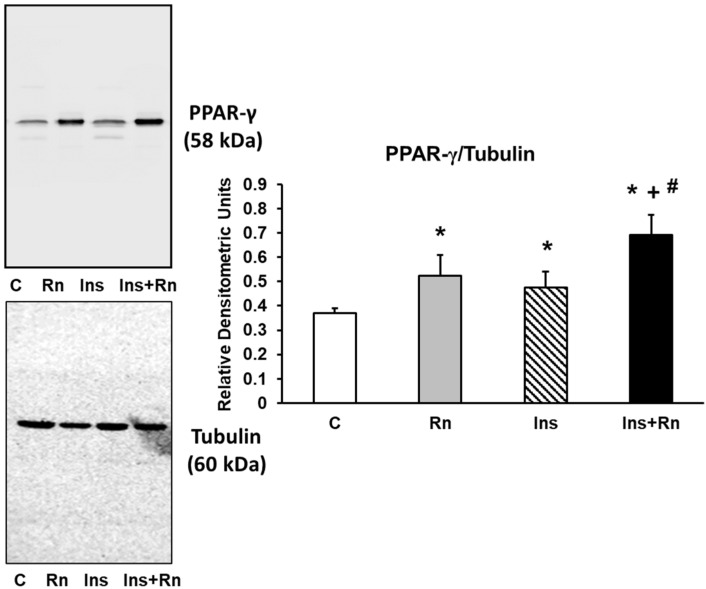
Effect of Ins and Rn on PPAR-γ protein expression. Astrocytes were incubated without Ins or Rn (control, C), with Rn (10^−6^ M), with Ins (10^−8^ M), or with Ins + Rn (10^−8^ M + 10^−6^ M) for 24 h and collected to determine PPAR-γ (1:300) and Tubulin (1:3000) protein expression by Western blot. A representative immunoblot is shown in the top panel. Data are mean ± SD of four independent experiments (four different rats). * *p* < 0.05 vs. control. + *p* < 0.05 vs. Rn. # *p* < 0.05 vs. Ins.

PPARs is a protein family that negatively regulates the gene expression of pro-inflammatory proteins. Figure 9 shows PPAR-γ expression in astrocytes in primary culture. Ins increased PPAR-γ expression compared to control astrocytes (46.8%). Furthermore, incubation with Ins + Rn increased PPAR-γ protein expression compared to control astrocytes (74.6%) and Ins-treated cells (18.4%) (Figure 9). 

## 3. Discussion

The main findings of this research are that Ins enhanced both cell viability and proliferation. Moreover, Ins increased p-AKT, p-eNOS, p-ERK, Mn-SOD, COX-2, and PPAR-γ protein expression in astrocytes in primary culture. Furthermore, Rn potentiated insulin-induced effects at doses like those seen in individuals treated with this medication. On the contrary, the expression of NF-κB and IκB after Rn, Ins, or Ins + Rn addition did not produce any alterations in astrocytes in the primary culture. The inclusion of Rn in the culture also resulted in a decrease in COX-2 protein expression. 

Astrocytes are glial cells that perform a variety of functions in the brain, including structural and metabolic support for the cell brain, maintenance of the blood–brain barrier [50], glutathione synthesis, and neuroprotective actions against oxidative stress and inflammation [2,51]. Astrocytes play a fundamental role in neuronal protection through a variety of mechanisms, the most notable of which is mitochondrial biogenesis, which allows them to shield neurons against inflammatory and oxidative processes [52].

Furthermore, astrocytes play roles in neuroendocrine, regulation of energy balance, and metabolism control by responding to the different hormonal stimuli [53,54]. Glucose uptake by astrocytes is an insulin-dependent process [55]. Astrocytes and microglia express insulin receptor isoforms as well as insulin receptor substrate (IRS)-1 and IRS-2 [56].

In our experiments, we found that Ins boosted the expression of p-AKT and p-eNOS. Functional studies with glial cells demonstrated that Ins activates PI3K and AKT [57]. Furthermore, AKT promotes NO production by mediating eNOS activation [58]. Insulin treatment of hippocampal CA1 cells improves memory and spatial learning. The synthesis of endogenous NO seems to be involved in these effects, since they are inhibited by L-NAME, a blocker of NO synthesis [59,60]. Insulin resistance appears to be implicated in cognitive decline in patients with type 2 diabetes (T2D) and Alzheimer’s disease. In addition, there is evidence that T2D and D1D (type 1 diabetes) patients show a higher frequency of depression, anxiety, cognitive impairment, and dementia [61,62].

A decrease in insulin release and/or a reduction in its sensitivity, are risk factors in both Alzheimer’s disease (AD) [63,64] and Parkinson’s disease (PD) [65]. Downregulation in the PI3K/AKT pathway is characteristic of insulin resistance [66]. Cognitive decline is associated with serine phosphorylation of IRS1 and co-localized with neurofibrillary tangles [67], decreasing insulin actions [68] by changes in the PI3K signaling pathway [69]. Furthermore, Rn causes a protective effect against cognitive decline in T2DM patients [70].

Insulin binding to its receptor activates the MAPK and ERK signaling pathways in addition to the AKT/eNOS pathway. ERK controls cell proliferation, mitogenesis, and differentiation, and the production of endothelin 1 [70]. Moreover, in the brain, insulin plays a key role in the direct regulation of ERK, which is involved in maintaining the type of memory involved in Alzheimer’s disease [71]. Our results show that insulin increases the expression of p-ERK, coinciding with the data presented by these authors.

Insulin inhibits the production of reactive oxygen species and iNOS expression when the cells are exposed to pro-inflammatory agents [72,73]. Furthermore, at low concentrations, insulin shows pro-inflammatory actions [56]. However, in our experiments, insulin did not show pro-inflammatory effects since there was no variation in the expression of NFκB and IκB and, on the contrary, it produced an overexpression of PPAR-γ. In diabetic patients and in animals with insulin resistance, PPARγ improves both glucose tolerance and cellular insulin sensitivity [74,75,76]. Moreover, insulin induces anti-inflammatory effects mediated by PPARγ and PI3K/Akt/Rac-1 signaling pathways [77]. In cardiovascular cells, activation of PPARγ inhibits the effects of angiotensin II and acts as an antioxidant and anti-inflammatory [78]. The use of PPARγ antagonists in neurodegenerative diseases associated with inflammatory processes has recently been proposed [79]. 

In our study, we observed that insulin caused an increase in the expression of COX-2. Insulin reduced amyloidogenesis and COX-2-mediated neuroinflammation in astrocytes treated with streptozotocin, which are hallmarks of Alzheimer’s disease [1]. On the contrary, intracerebral insulin administration decreased the expression of the inflammatory factor COX-2 in rats treated with streptozotocin [80].

In our experiments, insulin increased the expression of Mn-SOD and did not produce changes in Cu/Zn-SOD protein expression. In cardiomyocytes, the absence of insulin has been related to an increase in free radicals due to a decrease in SOD activity [81]. Insulin improves cognitive impairment in Wistar rats by reducing brain oxidative stress and increasing antioxidant systems such as SOD, catalase, and GSH [82]. Insulin resistance can be reversed with Mn-SOD mimetics or Mn-SOD overexpression [83]. In diabetic rats, insulin has been shown to protect against oxidative stress and inhibit apoptosis induced by H_2_O_2_ and intracellular ROS, and increase superoxide dismutase, catalase, and glutathione peroxidase activity [84]. 

Ranolazine improves ATP production and O2 consumption by stimulating glucose oxidation and decreasing fatty acid oxidation [85]. In type 2 diabetic patients, RN has been shown to offer a variety of effects, including lowering blood glucose and glycosylated haemoglobin levels, promoting insulin release, and decreasing glucagon synthesis, therefore improving pre- and postprandial blood glucose [86,87,88,89]. Rn reduced the pro-inflammatory profile and improved learning and long-term memory in a Wistar rat model of type 2 diabetes. Rn may be useful in addressing cognitive deterioration in type 2 diabetes in this way [69]. Its clinical use is especially interesting in patients with type 2 diabetes and coronary ischemia [32,90] and, in fact, Rn has been proposed as the first treatment for type 2 diabetes [88]. Rn does not modify the AKT pathway, or the kinases involved in glucose uptake [91]. In our experiments, Rn enhanced the effects of insulin on AKT and eNOS, increasing the expression of p-AKT and p-eNOS, indicating that this effect is probably due to a facilitation of insulin action.

The Rn improved insulin resistance in non-diabetic patients with coronary heart disease, reducing the homeostasis model assessment of insulin resistance (HOMA-IR) index with better results than that obtained with treatment with beta-blockers or calcium-channel blockers [92]. However, there is no direct evidence of the effects of Rn that increase cellular sensitivity to insulin. The data from our study seem to indicate a facilitating effect of Rn on the sensitivity of astrocytes to insulin.

Ranolazine interacts with different isoforms of the neuronal Nav channel [93], such as those involved in altered neuronal excitability in different forms of epilepsy, migraine, or neuropathic pain [94,95], which would allow its clinical use [35,94]. Moreover, Rn has recently been shown to improve diabetic neuropathy in rats [96]. Together, the cardioprotective and neuroprotective effects of Rn are related to its anti-inflammatory and antioxidant actions [4,97,98]. 

**Figure 10:**
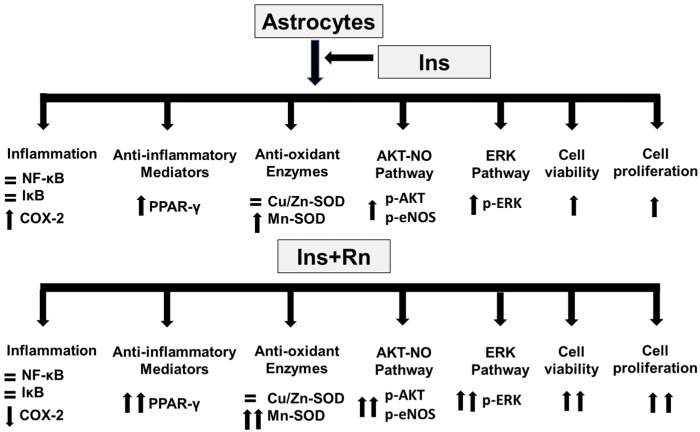
Changes after Ins and Rn addition to astrocytes in primary culture. Rn facilitates the effects of insulin, increasing cell viability and proliferation, along with the expression of anti-inflammatory mediators, such as PPAR-γ, and inhibiting that of pro-inflammatory mediators, such as COX-2. Furthermore, Rn potentiated the effect of insulin on the expression of an antioxidant enzyme (Mn-SOD), the components of the AKT-eNOS pathway, and the ERK signaling pathway.

In conclusion, ranolazine enhances the effects of insulin in primary culture astrocytes by boosting the expression of anti-inflammatory mediators such as PPAR-γ and reducing the production of pro-inflammatory mediators such as COX-2. Furthermore, ranolazine increased the action of insulin on the Mn-SOD antioxidant enzyme, as well as components of the AKT-eNOS and ERK signaling pathways (Figure 10).

Insulin resistance is the fundamental point in the pathophysiology of type 2 diabetes. Most of the treatments for this type of diabetes manage to increase insulin secretion by pancreatic β-cells, reducing blood glucose and glycosylated hemoglobin levels. However, these treatments have not shown efficacy in reducing insulin resistance or by increasing cellular sensitivity to the hormone. In our results, Rn produced a facilitation of the effects of insulin on nerve cells by increasing the sensitivity of astrocytes to the insulin. Therefore, ranolazine could be a useful drug to reverse peripheral insulin resistance. Moreover, type 3 diabetes has recently been described, in which it is proposed that neural resistance to insulin would be at the origin of Alzheimer’s disease, so ranolazine could also be a drug to be considered in the preventive treatment of Alzheimer’s disease.

## 4. Materials and Methods

### 4.1. Materials

3-(4,5-dimethyl-2-thiazolyl)-2,5-dipheniyl-2H tetrazolium bromide (MTT), ranolazine (Rn) (10^−6^ M), and insulin (Ins) (10^−8^ M) were obtained from Sigma-Aldrich biotechnology. anti-MnSOD (SAB2702309) (1:250), anti-NF-κB (MAB3026) (1:250), anti-IκB (ZRB1144) (1:250), anti-PPAR-γ (SAB4502262) (1:300), anti-COX-2 (SAB4503384) (1:500), anti-Cu/Zn-SOD (MABC684) (1:500), anti-AKT (SAB3701427) (1:500), anti-p-AKT (Ser473) (05-1003) (1:500), anti-eNOS (SAB5700744) (1:250), anti-phospho-eNOS (Ser1177) (07-428-I), anti-ERK1/2 (M5670) (1:500), anti-p-ERK1/2 (pThr202/Tyr204) (SAB4301578) (1:500), and anti-β-tubulin (T8328) (1:3000) antibodies (Sigma Aldrich, Madrid, Spain) were used. All other reagents were of analytical- or culture-grade purity.

### 4.2. Primary Culture of Cortical Astrocytes

All animals were handled according to the rules established by the bioethics committee of the School of Medicine, University of Valencia, Spain. Cerebral cortical astrocytes (from 21 days gestation) were obtained and plated on a T75 culture flask [4]. The medium was DMEM pH7.4, supplemented with 20% fetal bovine serum (FBS), 10 mM HEPES, 40 mM NaHCO3, 100 units/mL penicillin, and 100 mg/mL streptomycin.

The purity of astrocytes was assessed using anti-glial fibrillary acidic protein (anti-GFAP, astrocyte marker: Sigma-Aldrich, Madrid, Spain), anti-CD-68 (microglial marker: Serotec, Kidlington, UK), anti-myelin basic protein (oligodendroglial marker; Sigma-Aldrich, Madrid, Spain), and anti-microtubule-associated protein 2 (anti-MAP2, neuronal marker; Sigma-Aldrich, Madrid, Spain). The astrocytes were found to be at least 99% glial fibrillary acidic protein positive. No cells were found to express CD-68, myelin basic protein, or microtubule-associated protein-2. We used mitotracker to stain mitochondria, lysotracker to stain lysosomes, and Hoechst 33342 to stain nuclei. 

For all the experiments, we used toxin-free sterile culture materials.

### 4.3. MTT Assay

The cell viability of the cultures was determined by the MTT assay [99]. Astrocytes were plated in 96-well cultures. Rn, Ins, or Ins + Rn were added to wells for 24 h. After cell treatments, the medium was removed and the cortical cells were incubated with red free medium and MTT solution (0.5 mg/mL, prepared in a phosphate buffer saline (PBS) solution) for 4 h at 37 °C. Cell viability, defined as the relative amount of MTT reduction, was determined by spectrophotometry at 570 nm.

### 4.4. Trypan Blue Assay

A trypan blue exclusion assay was used to count the living cells and monitor cell proliferation. We applied 1.5% trypan blue solution to astrocyte cultures at room temperature for 3 min.

### 4.5. Western Blot Analysis

Protein concentration was determined using modified Lowry method [100]. After lysis, proteins were separated on SDS-PAGE gels and transferred to nitrocellulose membranes and incubated with primary antibodies overnight at 4 °C. Secondary anti-rabbit IgG or anti-mouse IgG (Cell Signaling Technologies Danvers, MA) antibody conjugated to the enzyme horseradish peroxidase (HRP) was used. Arbitrary units mean Relative Densitometric Units [4].

### 4.6. Statistical Methods

Values are expressed as mean ± S.D. Differences between groups were assessed using *t*-test (Student’s test) and by one-way analysis of variance (ANOVA) with the program GraphPad Prism. Statistical significance was accepted at *p* ≤ 0.05. Data sets in which F was significant were examined by a modified *t*-test.

## 5. Conclusions

Ranolazine enhances the effects of insulin in primary culture astrocytes by boosting the expression of anti-inflammatory mediators such as PPAR-γ and reducing the production of pro-inflammatory mediators such as COX-2. Furthermore, ranolazine increased the action of insulin on the Mn-SOD antioxidant enzyme, as well as components of the AKT-eNOS and ERK signaling pathways (Figure 10).

## Figures and Tables

**Table 1:** Effect of Ins and Rn on astrocytes’ proliferation. Cell proliferation and counting living cells. Astrocytes were isolated and seeded at 7 × 10^4^ cells/35 mm dish for 5 days. Cells were incubated without Ins or Rn (control, C), with Rn (10^−6^ M), with Ins (10^−8^ M), or with Ins + Rn (10^−8^ + 10^−6^ M) for 24 h. Trypan blue exclusion was used to count the living cells and monitor cell proliferation. Data are mean ± SD of four independent experiments (four different rats). * *p* < 0.05 vs. control. # *p* < 0.05 vs. Ins.

	Seeding Cells (×10^4^/35 mm Dish)	5 Days of Culture	24 h Treatment	% Proliferation
Control	7	12.86 ± 0.32	12.97 ± 0.24	0.85
Rn	7	12.87 ± 0.25	16.77 ± 0.35	30.31 *
Ins	7	12.85 ± 0.23	16.60 ± 0.37	29.18 *
Ins+Rn	7	12.88 ± 0.26	17.25 ± 0.35	33.91 *^,#^

## Data Availability

Not applicable.

## Abbreviations

Ins, insulin; Rn, ranolazine; AKT, protein kinase B; p-AKT, phosphor-protein kinase B; eNOS, endothelial nitric oxide synthase; p-eNOS, phosphor-endothelial nitric oxide synthase; ERK, extracellular-regulated kinase; p-ERK, phospho-extracellular-regulated kinase; COX-2, cyclooxygenase 2; Cu/Zn-SOD, Cu/Zn-superoxide dismutase; Mn-SOD, Mn-superoxide dismutase; NF-κB, nuclear factor-kappa B; IκB, an inhibitor of nuclear factor-kappa B; PPAR-γ, peroxisome proliferator-activated receptor γ.

## References

[1] Rajasekar N., Dwivedi S., Nath C., Hanif K., Shukla R. (2014). Protection of streptozotocin induced insulin receptor dysfunction, neuroinflammation and amyloidogenesis in astrocytes by insulin. Neuropharmacology.

[2] Ransohoff R.M., Engelhardt B. (2012). The anatomical and cellular basis of immune surveillance in the central nervous system. Nat. Rev. Immunol..

[3] Sofroniew M.V., Vinters H.V. (2010). Astrocytes: Biology and pathology. Acta Neuropathol..

[4] Aldasoro M., Guerra-Ojeda S., Aguirre-Rueda D., Mauricio M.D., Vila J.M., Marchio P., Iradi A., Aldasoro C., Jorda A., Obrador E. (2016). Effects of Ranolazine on Astrocytes and Neurons in Primary Culture. PLoS ONE.

[5] Thiebaud D., Jacot E., DeFronzo R.A., Maeder E., Jequier E., Felber J.P. (1982). The effect of graded doses of insulin on total glucose uptake, glucose oxidation, and glucose storage in man. Diabetes.

[6] Gray S.M., Barrett E.J. (2018). Insulin transport into the brain. American journal of physiology. Cell Physiol..

[7] Shaughness M., Acs D., Brabazon F., Hockenbury N., Byrnes K.R. (2020). Role of Insulin in Neurotrauma and Neurodegeneration: A Review. Front. Neurosci..

[8] Li H., Liu B., Huang J., Chen H., Guo X., Yuan Z. (2013). Insulin inhibits lipopolysaccharide-induced nitric oxide synthase expression in rat primary astrocytes. Brain Res..

[9] Heni M., Hennige A.M., Peter A., Siegel-Axel D., Ordelheide A.M., Krebs N., Machicao F., Fritsche A., Häring H.U., Staiger H. (2011). Insulin promotes glycogen storage and cell proliferation in primary human astrocytes. PLoS ONE.

[10] Shahriyary L., Riazi G., Lornejad M.R., Ghezlou M., Bigdeli B., Delavari B., Mamashli F., Abbasi S., Davoodi J., Saboury A.A. (2018). Effect of glycated insulin on the blood-brain barrier permeability: An in vitro study. Arch. Biochem. Biophys..

[11] Son S.M., Cha M.Y., Choi H., Kang S., Choi H., Lee M.S., Park S.A., Mook-Jung I. (2016). Insulin-degrading enzyme secretion from astrocytes is mediated by an autophagy-based unconventional secretory pathway in Alzheimer disease. Autophagy.

[12] Sousa L., Guarda M., Meneses M.J., Macedo M.P., Vicente Miranda H. (2021). Insulin-degrading enzyme: An ally against metabolic and neurodegenerative diseases. J. Pathol..

[13] Pellerin L., Magistretti P.J. (1994). Glutamate uptake into astrocytes stimulates aerobic glycolysis: A mechanism coupling neuronal activity to glucose utilization. Proc. Natl. Acad. Sci. USA.

[14] Magistretti P.J., Allaman I. (2018). Lactate in the brain: From metabolic end-product to signalling molecule. Nat. Rev. Neurosci..

[15] Arnold S.V., Echouffo-Tcheugui J.B., Lam C.S.P., Inzucchi S.E., Tang F., McGuire D.K., Goyal A., Maddox T.M., Sperling L.S., Fonarow G.C. (2018). Patterns of glucose-lowering medication use in patients with type 2 diabetes and heart failure. Insights from the Diabetes Collaborative Registry (DCR). Am. Heart J..

[16] Heni M., Eckstein S.S., Schittenhelm J., Böhm A., Hogrefe N., Irmler M., Beckers J., Hrabě de Angelis M., Häring H.U., Fritsche A. (2020). Ectopic fat accumulation in human astrocytes impairs insulin action. R. Soc. Open Sci..

[17] Clarke D.W., Boyd F.T., Kappy M.S., Raizada M.K. (1984). Insulin binds to specific receptors and stimulates 2-deoxy-D-glucose uptake in cultured glial cells from rat brain. J. Biol. Chem..

[18] Siddiqui M.A., Keam S.J. (2006). Ranolazine: A review of its use in chronic stable angina pectoris. Drugs.

[19] Storey K.M., Wang J., Garberich R.F., Bennett N.M., Traverse J.H., Arndt T.L., Schmidt C.W., Henry T.D. (2020). Long-Term (3 Years) Outcomes of Ranolazine Therapy for Refractory Angina Pectoris (from the Ranolazine Refractory Registry). Am. J. Cardiol..

[20] Stone P.H., Chaitman B.R., Stocke K., Sano J., DeVault A., Koch G.G. (2010). The anti-ischemic mechanism of action of ranolazine in stable ischemic heart disease. J. Am. Coll. Cardiol..

[21] Marchio P., Guerra-Ojeda S., Aldasoro M., Valles S.L., Martín-Gonzalez I., Martínez-León J.B., Mauricio M.D., Vila J.M. (2020). Relaxant and antiadrenergic effects of ranolazine in human saphenous vein. Eur. J. Cardio-Thorac. Surg..

[22] Nusca A., Bernardini F., Mangiacapra F., Maddaloni E., Melfi R., Ricottini E., Piccirillo F., Manfrini S., Ussia G.P., Grigioni F. (2021). Ranolazine Improves Glycemic Variability and Endothelial Function in Patients with Diabetes and Chronic Coronary Syndromes: Results from an Experimental Study. J. Diabetes Res..

[23] Shryock J.C., Belardinelli L. (2008). Inhibition of late sodium current to reduce electrical and mechanical dysfunction of ischaemic myocardium. Br. J. Pharmacol..

[24] Chaitman B.R. (2006). Ranolazine for the treatment of chronic angina and potential use in other cardiovascular conditions. Circulation.

[25] Belardinelli R., Lacalaprice F., Faccenda E., Volpe L. (2006). Clinical benefits of a metabolic approach in the cardiac rehabilitation of patients with coronary artery disease. Am. J. Cardiol..

[26] Kaplan A., Amin G., Abidi E., Altara R., Booz G.W., Zouein F.A. (2022). Role of ranolazine in heart failure: From cellular to clinic perspective. Eur. J. Pharmacol..

[27] Aldakkak M., Camara A.K., Heisner J.S., Yang M., Stowe D.F. (2011). Ranolazine reduces Ca2+ overload and oxidative stress and improves mitochondrial integrity to protect against ischemia reperfusion injury in isolated hearts. Pharmacol. Res..

[28] Rambarat C.A., Elgendy I.Y., Handberg E.M., Bairey Merz C.N., Wei J., Minissian M.B., Nelson M.D., Thomson L., Berman D.S., Shaw L.J. (2019). Late sodium channel blockade improves angina and myocardial perfusion in patients with severe coronary microvascular dysfunction: Women’s Ischemia Syndrome Evaluation-Coronary Vascular Dysfunction ancillary study. Int. J. Cardiol..

[29] Chou C.C., Lee H.L., Chang G.J., Wo H.T., Yen T.H., Wen M.S., Chu Y., Liu H.T., Chang P.C. (2020). Mechanisms of ranolazine pretreatment in preventing ventricular tachyarrhythmias in diabetic db/db mice with acute regional ischemia-reperfusion injury. Sci. Rep..

[30] Deshmukh S.H., Patel S.R., Pinassi E., Mindrescu C., Hermance E.V., Infantino M.N., Coppola J.T., Staniloae C.S. (2009). Ranolazine improves endothelial function in patients with stable coronary artery disease. Coron. Artery Dis..

[31] Morrow D.A., Scirica B.M., Chaitman B.R., McGuire D.K., Murphy S.A., Karwatowska-Prokopczuk E., McCabe C.H., Braunwald E., MERLIN-TIMI 36 Investigators (2019). Evaluation of the glycometabolic effects of ranolazine in patients with and without diabetes mellitus in the MERLIN-TIMI 36 randomized controlled trial. Circulation.

[32] Arnold S.V., McGuire D.K., Spertus J.A., Li Y., Yue P., Ben-Yehuda O., Belardinelli L., Jones P.G., Olmsted A., Chaitman B.R. (2014). Effectiveness of ranolazine in patients with type 2 diabetes mellitus and chronic stable angina according to baseline hemoglobin A1c. Am. Heart J..

[33] Koltun D.O., Parkhill E.Q., Elzein E., Kobayashi T., Jiang R.H., Li X., Perry T.D., Avila B., Wang W.Q., Hirakawa R. (2016). Discovery of triazolopyridinone GS-462808, a late sodium current inhibitor (Late INai) of the cardiac Nav1.5 channel with improved efficacy and potency relative to ranolazine. Bioorg. Med. Chem. Lett..

[34] Chunchai T., Arinno A., Ongnok B., Pantiya P., Khuanjing T., Prathumsap N., Maneechote C., Chattipakorn N., Chattipakorn S.C. (2022). Ranolazine alleviated cardiac/brain dysfunction in doxorubicin-treated rats. Exp. Mol. Pathol..

[35] Arnold S.V., Kosiborod M., McGuire D.K., Li Y., Yue P., Ben-Yehuda O., Spertus J.A. (2014). Effects of ranolazine on quality of life among patients with diabetes mellitus and stable angina. JAMA Intern. Med..

[36] Ning Y., Zhen W., Fu Z., Jiang J., Liu D., Belardinelli L., Dhalla A.K. (2011). Ranolazine increases β-cell survival and improves glucose homeostasis in low-dose streptozotocin-induced diabetes in mice. J. Pharmacol. Exp. Ther..

[37] Peters C.H., Sokolov S., Rajamani S., Ruben P.C. (2013). Effects of the antianginal drug, ranolazine, on the brain sodium channel Na(V)1.2 and its modulation by extracellular protons. Br. J. Pharmacol..

[38] Park Y.Y., Johnston D., Gray R. (2013). Slowly inactivating component of Na+ current in peri-somatic region of hippocampal CA1 pyramidal neurons. J. Neurophysiol..

[39] Virsolvy A., Farah C., Pertuit N., Kong L., Lacampagne A., Reboul C., Aimond F., Richard S. (2015). Antagonism of Nav channels and α1-adrenergic receptors contributes to vascular smooth muscle effects of ranolazine. Sci. Rep..

[40] Chen B.S., Lo Y.C., Peng H., Hsu T.I., Wu S.N. (2009). Effects of ranolazine, a novel anti-anginal drug, on ion currents and membrane potential in pituitary tumor GH (3) cells and NG108-15 neuronal cells. J. Pharmacol. Sci..

[41] Liao Y., Hung M.C. (2010). Physiological regulation of Akt activity and stability. Am. J. Transl. Res..

[42] Gerrits A.J., Koekman C.A., van Haeften T.W., Akkerman J.W. (2010). Platelet tissue factor synthesis in type 2 diabetic patients is resistant to inhibition by insulin. Diabetes.

[43] Niu W., Qi Y. (2011). An updated meta-analysis of endothelial nitric oxide synthase gene: Three well-characterized polymorphisms with hypertension. PLoS ONE.

[44] Shoukry A., Shalaby S.M., Abdelazim S., Abdelazim M., Ramadan A., Ismail M.I., Fouad M. (2012). Endothelial nitric oxide synthase gene polymorphisms and the risk of diabetic nephropathy in type 2 diabetes mellitus. Genet. Test. Mol. Biomark..

[45] Eröz R., Bahadir A., Dikici S., Tasdemir S. (2014). Association of endothelial nitric oxide synthase gene polymorphisms (894G/T, -786T/C, G10T) and clinical findings in patients with migraine. Neuromolecular Med..

[46] Kim S.J., Kahn C.R. (1997). Insulin regulation of mitogen-activated protein kinase kinase (MEK), mitogen-activated protein kinase and casein kinase in the cell nucleus: A possible role in the regulation of gene expression. Biochem. J..

[47] Tabatabaie T., Waldon A.M., Jacob J.M., Floyd R.A., Kotake Y. (2000). COX-2 inhibition prevents insulin-dependent diabetes in low-dose streptozotocin-treated mice. Biochem. Biophys. Res. Commun..

[48] McCord J.M., Fridovich I. (2014). Superoxide dismutases: You’ve come a long way, baby. Antioxid. Redox Signal..

[49] Muscogiuri G., Salmon A.B., Aguayo-Mazzucato C., Li M., Balas B., Guardado-Mendoza R., Giaccari A., Reddick R.L., Reyna S.M., Weir G. (2013). Genetic disruption of SOD1 gene causes glucose intolerance and impairs β-cell function. Diabetes.

[50] Karve I.P., Taylor J.M., Crack P.J. (2016). The contribution of astrocytes and microglia to traumatic brain injury. Br. J. Pharmacol..

[51] Skaper S.D. (2007). The brain as a target for inflammatory processes and neuroprotective strategies. Ann. N. Y. Acad. Sci..

[52] Aguirre-Rueda D., Guerra-Ojeda S., Aldasoro M., Iradi A., Obrador E., Ortega A., Mauricio M.D., Vila J.M., Valles S.L. (2015). Astrocytes protect neurons from Aβ1-42 peptide-induced neurotoxicity increasing TFAM and PGC-1 and decreasing PPAR-γ and SIRT-1. Int. J. Med. Sci..

[53] Bouyakdan K., Martin H., Liénard F., Budry L., Taib B., Rodaros D., Chrétien C., Biron É., Husson Z., Cota D. (2019). The gliotransmitter ACBP controls feeding and energy homeostasis via the melanocortin system. J. Clin. Investig..

[54] MacDonald A.J., Holmes F.E., Beall C., Pickering A.E., Ellacott K. (2020). Regulation of food intake by astrocytes in the brainstem dorsal vagal complex. Glia.

[55] González-García I., Gruber T., García-Cáceres C. (2021). Insulin action on astrocytes: From energy homeostasis to behaviour. J. Neuroendocrinol..

[56] Spielman L.J., Bahniwal M., Little J.P., Walker D.G., Klegeris A. (2015). Insulin Modulates In Vitro Secretion of Cytokines and Cytotoxins by Human Glial Cells. Curr. Alzheimer Res..

[57] Haas C.B., de Carvalho A.K., Muller A.P., Eggen B., Portela L.V. (2020). Insulin activates microglia and increases COX-2/IL-1β expression in young but not in aged hippocampus. Brain Res..

[58] Dimmeler S., Fleming I., Fisslthaler B., Hermann C., Busse R., Zeiher A.M. (1999). Activation of nitric oxide synthase in endothelial cells by Akt-dependent phosphorylation. Nature.

[59] Moosavi M., Naghdi N., Choopani S. (2007). Intra CA1 insulin microinjection improves memory consolidation and retrieval. Peptides.

[60] Choopani S., Moosavi M., Naghdi N. (2008). Involvement of nitric oxide in insulin induced memory improvement. Peptides.

[61] McCrimmon R.J., Ryan C.M., Frier B.M. (2012). Diabetes and cognitive dysfunction. Lancet.

[62] Nefs G., Hendrieckx C., Reddy P., Browne J.L., Bot M., Dixon J., Kyrios M., Speight J., Pouwer F. (2019). Comorbid elevated symptoms of anxiety and depression in adults with type 1 or type 2 diabetes: Results from the International Diabetes MILES Study. J. Diabetes Its Complicat..

[63] Arvanitakis Z., Wilson R.S., Bienias J.L., Evans D.A., Bennett D.A. (2004). Diabetes mellitus and risk of Alzheimer disease and decline in cognitive function. Arch. Neurol..

[64] Akhtar A., Sah S.P. (2020). Insulin signaling pathway and related molecules: Role in neurodegeneration and Alzheimer’s disease. Neurochem. Int..

[65] Cheong J., de Pablo-Fernandez E., Foltynie T., Noyce A.J. (2020). The Association Between Type 2 Diabetes Mellitus and Parkinson’s Disease. J. Parkinson’s Dis..

[66] Gabbouj S., Ryhänen S., Marttinen M., Wittrahm R., Takalo M., Kemppainen S., Martiskainen H., Tanila H., Haapasalo A., Hiltunen M. (2019). Altered Insulin Signaling in Alzheimer’s Disease Brain - Special Emphasis on PI3K-Akt Pathway. Front. Neurosci..

[67] Talbot K., Wang H.Y., Kazi H., Han L.Y., Bakshi K.P., Stucky A., Fuino R.L., Kawaguchi K.R., Samoyedny A.J., Wilson R.S. (2012). Demonstrated brain insulin resistance in Alzheimer’s disease patients is associated with IGF-1 resistance, IRS-1 dysregulation, and cognitive decline. J. Clin. Investig..

[68] Yarchoan M., Toledo J.B., Lee E.B., Arvanitakis Z., Kazi H., Han L.Y., Louneva N., Lee V.M., Kim S.F., Trojanowski J.Q. (2014). Abnormal serine phosphorylation of insulin receptor substrate 1 is associated with tau pathology in Alzheimer’s disease and tauopathies. Acta Neuropathol..

[69] Metz H.E., Houghton A.M. (2011). Insulin receptor substrate regulation of phosphoinositide 3-kinase. Clin. Cancer Res..

[70] Cassano V., Leo A., Tallarico M., Nesci V., Cimellaro A., Fiorentino T.V., Citraro R., Hribal M.L., De Sarro G., Perticone F. (2020). Metabolic and Cognitive Effects of Ranolazine in Type 2 Diabetes Mellitus: Data from an in vivo Model. Nutrients.

[71] Marasciulo F.L., Montagnani M., Potenza M.A. (2006). Endothelin-1: The yin and yang on vascular function. Curr. Med. Chem..

[72] Lee J.H., Jahrling J.B., Denner L., Dineley K.T. (2018). Targeting Insulin for Alzheimer’s Disease: Mechanisms, Status and Potential Directions. J. Alzheimer’s Dis..

[73] Brabazon F., Bermudez S., Shaughness M., Khayrullina G., Byrnes K.R. (2018). The effects of insulin on the inflammatory activity of BV2 microglia. PLoS ONE.

[74] Picard F., Auwerx J. (2002). PPAR(gamma) and glucose homeostasis. Annu. Rev. Nutr..

[75] Norris A.W., Chen L., Fisher S.J., Szanto I., Ristow M., Jozsi A.C., Hirshman M.F., Rosen E.D., Goodyear L.J., Gonzalez F.J. (2003). Muscle-specific PPARgamma-deficient mice develop increased adiposity and insulin resistance but respond to thiazolidinediones. J. Clin. Investig..

[76] Montaigne D., Butruille L., Staels B. (2021). PPAR control of metabolism and cardiovascular functions. Nature reviews. Cardiology.

[77] Yu T., Gao M., Yang P., Liu D., Wang D., Song F., Zhang X., Liu Y. (2019). Insulin promotes macrophage phenotype transition through PI3K/Akt and PPAR-γ signaling during diabetic wound healing. J. Cell. Physiol..

[78] Touyz R.M., Schiffrin E.L. (2006). Peroxisome proliferator-activated receptors in vascular biology-molecular mechanisms and clinical implications. Vasc. Pharmacol..

[79] Lee Y., Cho J.H., Lee S., Lee W., Chang S.C., Chung H.Y., Moon H.R., Lee J. (2019). Neuroprotective effects of MHY908, a PPAR α/γ dual agonist, in a MPTP-induced Parkinson’s disease model. Brain Res..

[80] Rajasekar N., Nath C., Hanif K., Shukla R. (2017). Intranasal Insulin Administration Ameliorates Streptozotocin (ICV)-Induced Insulin Receptor Dysfunction, Neuroinflammation, Amyloidogenesis, and Memory Impairment in Rats. Mol. Neurobiol..

[81] Matsuzaki S., Eyster C., Newhardt M.F., Giorgione J.R., Kinter C., Young Z.T., Kinter M., Humphries K.M. (2021). Insulin signaling alters antioxidant capacity in the diabetic heart. Redox Biol..

[82] Ramalingayya G.V., Sonawane V., Cheruku S.P., Kishore A., Nayak P.G., Kumar N., Shenoy R.S., Nandakumar K. (2017). Insulin Protects against Brain Oxidative Stress with an Apparent Effect on Episodic Memory in Doxorubicin-Induced Cognitive Dysfunction in Wistar Rats. J. Environ. Pathol. Toxicol. Oncol..

[83] Hoehn K.L., Salmon A.B., Hohnen-Behrens C., Turner N., Hoy A.J., Maghzal G.J., Stocker R., Van Remmen H., Kraegen E.W., Cooney G.J. (2009). Insulin resistance is a cellular antioxidant defense mechanism. Proc. Natl. Acad. Sci. USA.

[84] Song Y., Ding W., Bei Y., Xiao Y., Tong H.D., Wang L.B., Ai L.Y. (2018). Insulin is a potential antioxidant for diabetes-associated cognitive decline via regulating Nrf2 dependent antioxidant enzymes. Biomed. Pharmacother..

[85] McCormack J.G., Barr R.L., Wolff A.A., Lopaschuk G.D. (1996). Ranolazine stimulates glucose oxidation in normoxic, ischemic, and reperfused ischemic rat hearts. Circulation.

[86] Fu Z., Zhao L., Chai W., Dong Z., Cao W., Liu Z. (2013). Ranolazine recruits muscle microvasculature and enhances insulin action in rats. J. Physiol..

[87] Zeng X., Zhang Y., Lin J., Zheng H., Peng J., Huang W. (2017). Efficacy and Safety of Ranolazine in Diabetic Patients: A Systematic Review and Meta-analysis. Ann. Pharmacother..

[88] Gilbert B.W., Sherard M., Little L., Branstetter J., Meister A., Huffman J. (2018). Antihyperglycemic and Metabolic Effects of Ranolazine in Patients with Diabetes Mellitus. Am. J. Cardiol..

[89] Bell D., Goncalves E. (2021). Diabetogenic effects of cardioprotective drugs. Diabetes Obes. Metab..

[90] Teoh I.H., Banerjee M. (2018). Effect of ranolazine on glycaemia in adults with and without diabetes: A meta-analysis of randomised controlled trials. Open Heart.

[91] Terruzzi I., Montesano A., Senesi P., Vacante F., Benedini S., Luzi L. (2017). Ranolazine promotes muscle differentiation and reduces oxidative stress in C2C12 skeletal muscle cells. Endocrine.

[92] Caminiti G., Fossati C., Battaglia D., Massaro R., Rosano G., Volterrani M. (2016). Ranolazine improves insulin resistance in non-diabetic patients with coronary heart disease. A pilot study. Int. J. Cardiol..

[93] Theile J.W., Cummins T.R. (2011). Recent developments regarding voltage-gated sodium channel blockers for the treatment of inherited and acquired neuropathic pain syndromes. Front. Pharmacol..

[94] Kahlig K.M., Lepist I., Leung K., Rajamani S., George A.L. (2010). Ranolazine selectively blocks persistent current evoked by epilepsy-associated Naν1.1 mutations. Br. J. Pharmacol..

[95] Nodera H., Rutkove S.B. (2012). Changes of the peripheral nerve excitability in vivo induced by the persistent Na+ current blocker ranolazine. Neurosci. Lett..

[96] Elkholy S.E., Elaidy S.M., El-Sherbeeny N.A., Toraih E.A., El-Gawly H.W. (2020). Neuroprotective effects of ranolazine versus pioglitazone in experimental diabetic neuropathy: Targeting Nav1.7 channels and PPAR-γ. Life Sci..

[97] Rouhana S., Virsolvy A., Fares N., Richard S., Thireau J. (2021). Ranolazine: An Old Drug with Emerging Potential; Lessons from Pre-Clinical and Clinical Investigations for Possible Repositioning. Pharmaceuticals.

[98] Efentakis P., Andreadou I., Bibli S.I., Vasileiou S., Dagres N., Zoga A., Lougiakis N., Kremastinos D.T., Iliodromitis E.K. (2016). Ranolazine triggers pharmacological preconditioning and postconditioning in anesthetized rabbits through activation of RISK pathway. Eur. J. Pharmacol..

[99] Stockert J.C., Blázquez-Castro A., Cañete M., Horobin R.W., Villanueva A. (2012). MTT assay for cell viability: Intracellular localization of the formazan product is in lipid droplets. Acta Histochemical..

[100] Barja G. (1999). Mitochondrial oxygen radical generation and leak: Sites of production in states 4 and 3, organ specificity, and relation to aging and longevity. J. Bioenerg. Biomembr..

